# Abnormalities in synaptic dynamics during development in a mouse model of spinocerebellar ataxia type 1

**DOI:** 10.1038/srep16102

**Published:** 2015-11-04

**Authors:** Yusuke Hatanaka, Kei Watase, Keiji Wada, Yoshitaka Nagai

**Affiliations:** 1 Department of Degenerative Neurological Diseases, National Institute of Neuroscience, National Center of Neurology and Psychiatry, 4-1-1 Ogawa-Higashi, Kodaira, Tokyo 187-8502, Japan; 2 CREST, JST, 4-1-8 Honcho, Kawaguchi, Saitama, 332-0012, Japan; 3 Center for Brain Integration Research, Tokyo Medical & Dental University, 1-5-45 Yushima, Bunkyo, Tokyo 113-8510, Japan

## Abstract

Late-onset neurodegenerative diseases are characterized by neurological symptoms and progressive neuronal death. Accumulating evidence suggests that neuronal dysfunction, rather than neuronal death, causes the symptoms of neurodegenerative diseases. However, the mechanisms underlying the dysfunction that occurs prior to cell death remain unclear. To investigate the synaptic basis of this dysfunction, we employed *in vivo* two-photon imaging to analyse excitatory postsynaptic dendritic protrusions. We used *Sca1*^*154Q*/*2Q*^ mice, an established knock-in mouse model of the polyglutamine disease spinocerebellar ataxia type 1 (SCA1), which replicates human SCA1 features including ataxia, cognitive impairment, and neuronal death. We found that *Sca1*^*154Q*/*2Q*^ mice exhibited greater synaptic instability than controls, without synaptic loss, in the cerebral cortex, where obvious neuronal death is not observed, even before the onset of distinct symptoms. Interestingly, this abnormal synaptic instability was evident in *Sca1*^*154Q*/*2Q*^ mice from the synaptic developmental stage, and persisted into adulthood. Expression of synaptic scaffolding proteins was also lower in *Sca1*^*154Q*/*2Q*^ mice than controls before synaptic maturation. As symptoms progressed, synaptic loss became evident. These results indicate that aberrant synaptic instability, accompanied by decreased expression of scaffolding proteins during synaptic development, is a very early pathology that precedes distinct neurological symptoms and neuronal cell death in SCA1.

Many late-onset neurodegenerative diseases, including Alzheimer’s, Parkinson’s, and prion and polyglutamine diseases, share common features, such as the aggregation of toxic proteins in neurons, and progressive neuronal cell death^1^,^2^. Accumulating evidence from patients and animal models suggests that the initial symptoms of neurodegenerative diseases are a result of neuronal dysfunction rather than cell death^3^. However, the nature of this dysfunction that occurs prior to cell death remains unknown.

Many neurodegenerative diseases are attributed to multiple factors, including genetic and environmental predispositions. On the other hand, spinocerebellar ataxia type 1 (SCA1), a polyglutamine disease, is a monogenic disorder caused by the expansion of an unstable CAG trinucleotide repeat tract encoding a polyglutamine stretch in the *ATXN1* gene^4^. The *Sca1*^*154Q*/*2Q*^ knock-in mouse model, harbouring 154 CAG repeats within the endogenous *ATXN1* locus, closely reproduces the features of human SCA1^5^, including neuronal cell death, ataxia, motor incoordination, and cognitive impairment^6^,^7^. Although the number of CAG repeats in *Sca1*^*154Q*/*2Q*^ mice is much higher than that in human patients, another knock-in SCA1 mouse model harbouring 78 CAG repeats, similar to the number in patients, displays only mild behavioural deficits late in life^8^. Thus, *Sca1*^*154Q*/*2Q*^ mice are suitable for studying symptom progression. *Sca1*^*154Q*/*2Q*^ mice develop motor learning impairment before any obvious Purkinje cell death occurs or nuclear inclusions form in the cerebellum^5^. In the limbic area, *Sca1*^*154Q*/*2Q*^ mice show nuclear inclusions in pyramidal neurons, and cognitive deficits are observed without evident neuronal loss^5^. Clinical studies have demonstrated that neuronal death is most prominent in the cerebellum, whereas little occurs in the cerebral cortex and hippocampus, despite the presence of cognitive impairments in patients with SCA1^6^. These lines of evidence suggest that neuronal dysfunction, preceding cell death, causes subsequent behavioural impairments in the pathogenesis of SCA1; however, the mechanisms underlying the dysfunction remain unclear.

In the present study, we focused on SCA1 as a genetic model of neurodegenerative disease, and used *Sca1*^*154Q*/*2Q*^ knock-in mice to elucidate the synaptic basis of neuronal dysfunction. We analysed the dynamics, morphology, and density of dendritic protrusions, which are excitatory postsynaptic structures classified into mature ‘spines’ and immature ‘filopodia’. These features are strongly associated with synaptic development^9^, plasticity^10^, and various pathologies^11^. Using two-photon laser-scanning microscopy, we investigated the synaptic pathologies of *Sca1*^*154Q*/*2Q*^ knock-in mice *in vivo*, maintaining contributions from peripheral tissues and non-neuronal cells expressing mutant ataxin-1, as well as neurons. To evaluate neuronal dysfunction while excluding the effects of neuronal death, we focused on the cerebral cortex and hippocampus, in which apparent neuronal death does not occur despite the presence of cognitive dysfunction in both *Sca1*^*154Q*/*2Q*^ mice and human SCA1 patients^5^,^7^. Our findings demonstrate that aberrant synaptic instability accompanied by a reduction in the expression of scaffolding proteins in affected neurons appears during synaptic development in SCA1 mice. These results suggest that deficits in neuronal circuitry development may underlie subsequent behavioural and neurological impairments in late-onset neurodegenerative diseases.

## Results

### SCA1 mice show aberrant instability of dendritic protrusions before the onset of distinct symptoms

**Figure 1:**
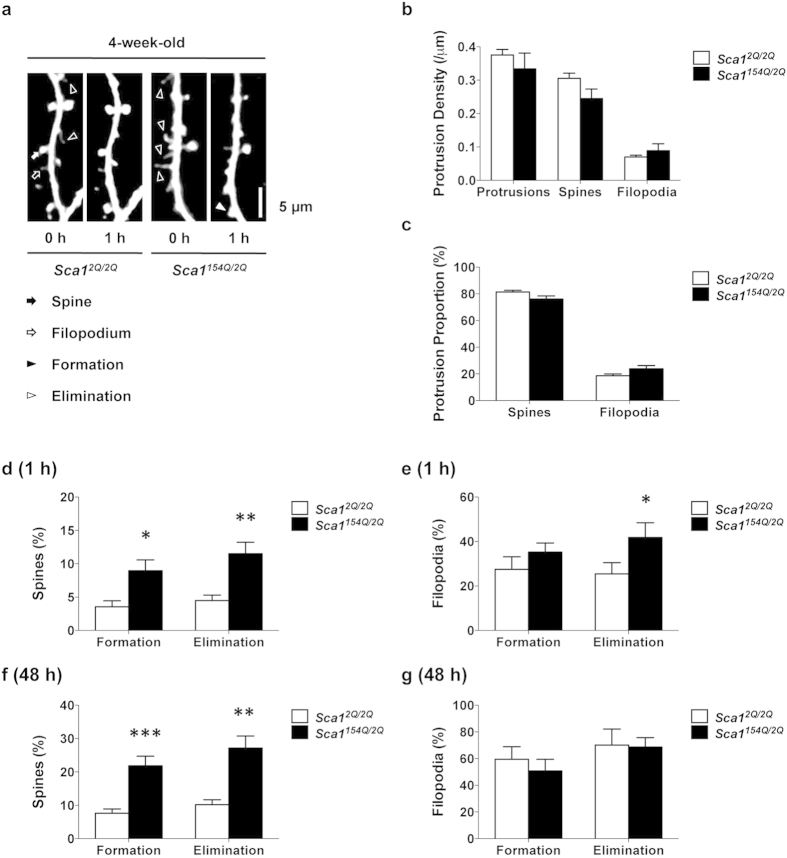
SCA1 mice show abnormal synaptic instability before the onset of distinct symptoms. (**a**) *In vivo* two-photon imaging of layer 1 dendrites of layer 5 pyramidal neurons in 4-week-old *Sca1*^*2Q*/*2Q*^ mice (*n* = 14 dendrites from five animals) and *Sca1*^*154Q*/*2Q*^ mice (*n* = 17 dendrites from five animals). Repeated imaging of the same dendrites in each group over 1 h enables visualization of the formation (filled arrowhead) and elimination (open arrowheads) of dendritic protrusions in *Sca1*^*154Q*/*2Q*^ mice at 4 weeks of age. Dendritic protrusions were classified into two groups: spines (filled arrow) and filopodia (open arrow). Images are best projections (3–7 optical sections, 0.75 μm apart). (**b**) Dendritic protrusion density in 4-week-old *Sca1*^*2Q*/*2Q*^ and *Sca1*^*154Q*/*2Q*^ mice. (**c**) Spines and filopodia as a percentage of total protrusions in *Sca1*^*2Q*/*2Q*^ and *Sca1*^*154Q*/*2Q*^ mice. (**d**) Percentage of total spines formed and eliminated over 1 h. *Sca1*^*154Q*/*2Q*^ mice at 4 weeks of age showed higher formation and elimination rates of spines than *Sca1*^*2Q*/*2Q*^ mice. (**e**) Percentage of total filopodia formed and eliminated over 1 h. In 4-week-old *Sca1*^*154Q*/*2Q*^ mice, the elimination rate of filopodia was higher than that in *Sca1*^*2Q*/*2Q*^ mice, whereas the rate of formation was not different from *Sca1*^*2Q*/*2Q*^ mice. (**f**) Percentage of total spines formed and eliminated over 48 h. *Sca1*^*154Q*/*2Q*^ mice at 4 weeks of age (*n* = 12 dendrites from six mice) exhibited higher formation and elimination rates of spines than *Sca1*^*2Q*/*2Q*^ mice (*n* = 18 dendrites from eight mice). (**g**) Percentage of total filopodia formed and eliminated over 48 h. No differences were observed in the rates of formation or elimination of filopodia between 4-week-old *Sca1*^*2Q*/*2Q*^ and *Sca1*^*154Q*/*2Q*^ mice. Data are presented as the mean ± SEM. *p < 0.05, **p < 0.01, Student *t*-test. Scale bar, 5 μm.

Motor learning impairments in *Sca1*^*154Q*/*2Q*^ mice are observed by 5 weeks of age, and spatial and fear memory deficits by 8 weeks. Although nuclear inclusions of mutant ataxin-1 are observed by 6 weeks, there is no neuronal death in the limbic area during such early stages of the disease^5^. We therefore investigated synaptic abnormalities in 4-week-old *Sca1*^*154Q*/*2Q*^ mice as a possible early SCA1 phenotype. We performed *in vivo* two-photon imaging in layer 1 dendrites of the primary somatosensory cortex in *Sca1*^*154Q*/*2Q*^ and control *Sca1*^*2Q*/*2Q*^ mice, and analysed the morphology, formation, and elimination of dendritic protrusions over a 1 h period under anaesthesia (Fig. 1a). Dendritic protrusions were classified into spines and filopodia according to their morphology, because filopodia are less stable than spines, and their density decreases with development^9^. We did not observe any clear differences between *Sca1*^*2Q*/*2Q*^ and *Sca1*^*154Q*/*2Q*^ mice in the morphology of dendritic protrusions. Furthermore, we found no significant differences in the density of protrusions between *Sca1*^*2Q*/*2Q*^ and *Sca1*^*154Q*/*2Q*^ mice at 4 weeks of age (Fig. 1b) [total protrusions: *Sca1*^*2Q*/*2Q*^ (0.38 ± 0.02/μm) vs *Sca1*^*154Q*/*2Q*^ (0.33 ± 0.05/μm), *p* = 0.4532; spines: *Sca1*^*2Q*/*2Q*^ (0.31 ± 0.02/μm) vs *Sca1*^*154Q*/*2Q*^ (0.24 ± 0.03/μm), *p* = 0.0909; filopodia: *Sca1*^*2Q*/*2Q*^ (0.070 ± 0.005/μm) vs *Sca1*^*154Q*/*2Q*^ (0.09 ± 0.02/μm), *p* = 0.4159; unpaired *t*-test]. There were also no significant differences between 4-week-old *Sca1*^*2Q*/*2Q*^ and *Sca1*^*154Q*/*2Q*^ mice in the number of spines or filopodia as a percentage of the total protrusions (Fig. 1c) [spines: *Sca1*^*2Q*/*2Q*^ (81 ± 1%) vs *Sca1*^*154Q*/*2Q*^ (76 ± 2%), *p* = 0.0747; filopodia: *Sca1*^*2Q*/*2Q*^ (19 ± 1%) vs *Sca1*^*154Q*/*2Q*^ (24 ± 2%), *p* = 0.0747; unpaired *t*-test]. Next, we analysed the dynamics of the protrusions in order to estimate synaptic stability. Notably, we found that the rates of formation and elimination of spines over 1 h were significantly higher in *Sca1*^*154Q*/*2Q*^ mice than in *Sca1*^*2Q*/*2Q*^ mice (Fig. 1d) [formation rate: *Sca1*^*2Q*/*2Q*^ (3.6 ± 0.9%) vs *Sca1*^*154Q*/*2Q*^ (9 ± 2%), *p* = 0.0101; elimination rate: *Sca1*^*2Q*/*2Q*^ (4.5 ± 0.8%) vs *Sca1*^*154Q*/*2Q*^ (12 ± 2%), *p* = 0.0017; unpaired *t*-test]. The elimination rate of filopodia over 1 h was also significantly higher in *Sca1*^*154Q*/*2Q*^ mice, but the difference in their formation rate did not reach statistical significance (Fig. 1e) [formation rate: *Sca1*^*2Q*/*2Q*^ (27 ± 6%) vs *Sca1*^*154Q*/*2Q*^ (35 ± 4%), *p* = 0.2711; elimination rate: *Sca1*^*2Q*/*2Q*^ (23 ± 4%) vs *Sca1*^*154Q*/*2Q*^ (42 ± 7%), *p* = 0.0341; unpaired *t*-test]. Anaesthetics can increase the formation of filopodia within a 1 h period^12^; therefore, to eliminate the effects of anaesthesia on synaptic dynamics, we performed *in vivo* imaging over 48 h, during which time the mice were allowed to recover from the anaesthesia after the first imaging session and were returned to their home cages until the next session. We confirmed that both formation and elimination rates of spines in *Sca1*^*154Q*/*2Q*^ mice were also significantly higher than those in *Sca1*^*2Q*/*2Q*^ mice over a 48 h period (Fig. 1f) [formation rate: *Sca1*^*2Q*/*2Q*^ (8 ± 1%) vs *Sca1*^*154Q*/*2Q*^ (23 ± 3%), *p* < 0.0001; elimination rate: *Sca1*^*2Q*/*2Q*^ (10 ± 1%) vs *Sca1*^*154Q*/*2Q*^ (28 ± 4%), *p* = 0.0003; unpaired *t*-test]. Neither the formation nor elimination rates of filopodia over 48 h were significantly different between groups, probably owing to the very high rates measured over 48 h even in control mice (Fig. 1g) [formation rate: *Sca1*^*2Q*/*2Q*^ (59 ± 9%) vs *Sca1*^*154Q*/*2Q*^ (52 ± 9%), *p* = 0.5763; elimination rate: *Sca1*^*2Q*/*2Q*^ (70 ± 10%) vs *Sca1*^*154Q*/*2Q*^ (72 ± 7%), *p* = 0.8919; unpaired *t*-test]. These data indicate that *Sca1*^*154Q*/*2Q*^ mice show abnormal synaptic instability during synaptic development, before the onset of obvious symptoms, and that these altered dynamics are detectable by imaging under anaesthesia during a 1 h period.

### SCA1 mice develop abnormal protrusion morphology with persisting synaptic instability

**Figure 2:**
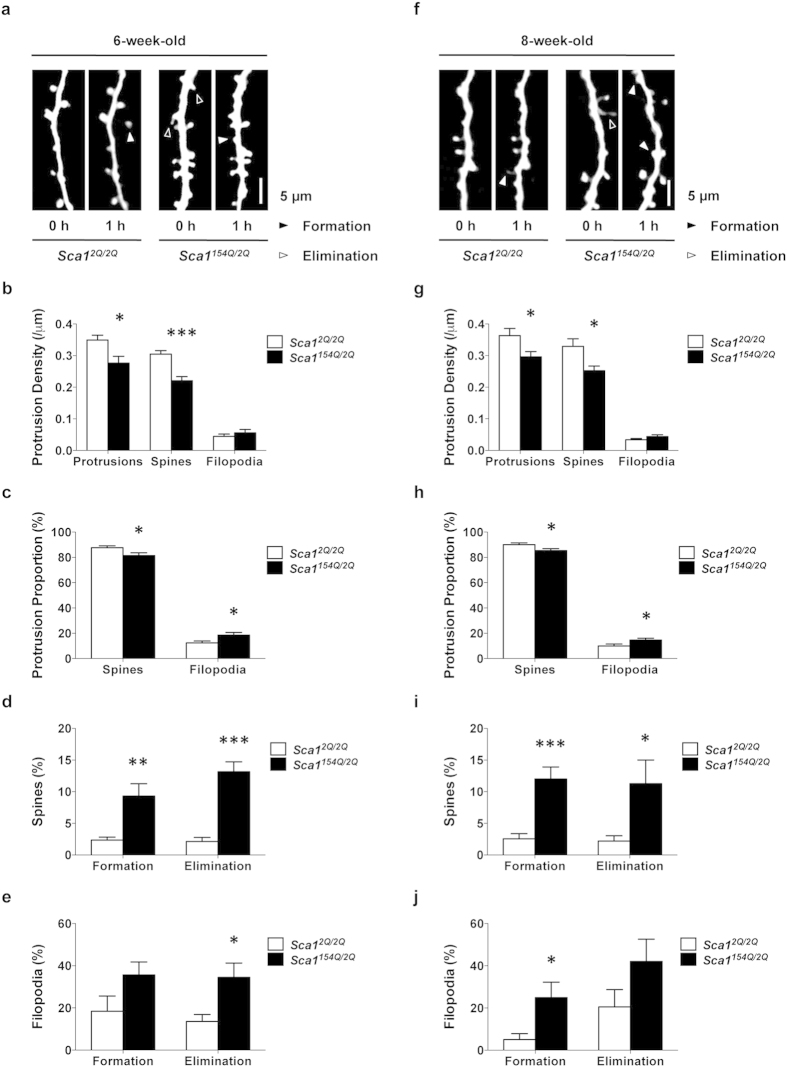
SCA1 mice develop immature protrusion morphology and loss of dendritic protrusions, associated with persisting synaptic instability after symptom onset. (**a**,**f**) *In vivo* two-photon imaging of dendrites in 6- and 8-week-old *Sca1*^*2Q*/*2Q*^ mice (*n* = 14 dendrites from five mice and *n* = 12 dendrites from four mice, respectively) and *Sca1*^*154Q*/*2Q*^ mice (*n* = 14 dendrites from five mice and *n* = 10 dendrites from five mice, respectively). Repeated imaging of the same dendrites over 1 h in each group showed increased formation (filled arrowhead) and elimination (open arrowheads) of dendritic protrusions in *Sca1*^*154Q*/*2Q*^ mice at 6 and 8 weeks of age. (**b**,**g**) Dendritic protrusion density in 6- and 8-week-old *Sca1*^*2Q*/*2Q*^ and *Sca1*^*154Q*/*2Q*^ mice. *Sca1*^*154Q*/*2Q*^ mice exhibited decreased spine density at both 6 (**b**) and 8 (**g**) weeks of age. (**c**,**h**) Spines and filopodia as a percentage of total protrusions in *Sca1*^*2Q*/*2Q*^ and *Sca1*^*154Q*/*2Q*^ mice at 6 (**c**) and 8 (**h**) weeks of age. The percentage of spines was lower, and filopodia higher, in 8-week-old *Sca1*^*154Q*/*2Q*^ mice than in *Sca1*^*2Q*/*2Q*^ mice, whereas no differences were observed in 6-week-old *Sca1*^*154Q*/*2Q*^ mice. (**d**,**i**) Percentage of total spines formed and eliminated. *Sca1*^*154Q*/*2Q*^ mice showed higher formation and elimination rates of spines than *Sca1*^*2Q*/*2Q*^ mice at 6 (**d**) and 8 (**i**) weeks of age. (**e**,**j**) Percentage of total filopodia formed and eliminated. Formation and elimination rates were greater in 6- (**f**) and 8-week-old *Sca1*^*154Q*/*2Q*^ mice than in *Sca1*^*2Q*/*2Q*^ mice (**k**). Data are presented as the mean ± SEM. *p < 0.05, **p < 0.01, ***p < 0.001, Student *t*-test. Scale bar, 5 μm.

We demonstrated that synaptic instability occurs in SCA1 mice before the onset of distinct symptoms at 4 weeks of age. Next, we evaluated the progression of synaptic pathology in SCA1 mice until 8 weeks of age, by which time dendritic spines have normally stabilised and the density of filopodia has reached a minimum^9^. *Sca1*^*154Q*/*2Q*^ mice develop motor learning impairment and nuclear inclusions by 6 weeks of age, and memory deficits by 8 weeks^5^. We investigated the dynamics and morphology of dendritic protrusions in *Sca1*^*2Q*/*2Q*^ and *Sca1*^*154Q*/*2Q*^ mice at 6 (Fig. 2a) and 8 weeks of age (Fig. 2f). In 6-week-old *Sca1*^*154Q*/*2Q*^ mice, the density of dendritic spines and protrusions was significantly lower than that in age-matched *Sca1*^*2Q*/*2Q*^ mice, whereas the density of filopodia did not differ between the groups (Fig. 2b) [protrusions: *Sca1*^*2Q*/*2Q*^ (0.35 ± 0.02/μm) vs *Sca1*^*154Q*/*2Q*^ (0.28 ± 0.02/μm), *p* = 0.0101; spines: *Sca1*^*2Q*/*2Q*^ (0.30 ± 0.01/μm) vs *Sca1*^*154Q*/*2Q*^ (0.22 ± 0.01/μm), *p* < 0.0001; filopodia: *Sca1*^*2Q*/*2Q*^ (0.04 ± 0.01/μm) vs *Sca1*^*154Q*/*2Q*^ (0.06 ± 0.01/μm), *p* = 0.3871; unpaired *t*-test]. The number of spines as a percentage of total protrusions was significantly lower in *Sca1*^*154Q*/*2Q*^ mice than in *Sca1*^*2Q*/*2Q*^ mice at 6 weeks (Fig. 2c) [*Sca1*^*2Q*/*2Q*^ (88 ± 2%) vs *Sca1*^*154Q*/*2Q*^ (81 ± 2%), *p* = 0.0284; unpaired *t*-test], whereas that of filopodia was higher (Fig. 2c) [*Sca1*^*2Q*/*2Q*^ (12 ± 2% vs *Sca1*^*154Q*/*2Q*^ (19 ± 2%), *p* = 0.0284; unpaired *t*-test]. Spine dynamics were significantly greater in *Sca1*^*154Q*/*2Q*^ mice than in *Sca1*^*2Q*/*2Q*^ mice at 6 weeks of age (Fig. 2d) [formation rate: *Sca1*^*2Q*/*2Q*^ (2.3 ± 0.5%) vs *Sca1*^*154Q*/*2Q*^ (9 ± 2%), *p* = 0.0018; elimination rate: *Sca1*^*2Q*/*2Q*^ (2.1 ± 0.6%) vs *Sca1*^*154Q*/*2Q*^ (13 ± 2%), *p* < 0.0001; unpaired *t*-test]. Regarding the filopodium dynamics of 6-week-old *Sca1*^*154Q*/*2Q*^ mice, the elimination rate was significantly higher, but the formation rate was not different from that in *Sca1*^*2Q*/*2Q*^ mice (Fig. 2e) [formation rate: *Sca1*^*2Q*/*2Q*^ (18 ± 7%) vs *Sca1*^*154Q*/*2Q*^ (36 ± 6%), *p* = 0.0785; elimination rate: *Sca1*^*2Q*/*2Q*^ (14 ± 3%) vs *Sca1*^*154Q*/*2Q*^ (35 ± 7%), *p* = 0.0100; unpaired *t*-test]. In 8-week-old *Sca1*^*154Q*/*2Q*^ mice, total protrusion and spine densities were significantly lower than those in age-matched *Sca1*^*2Q*/*2Q*^ mice, whereas filopodium density was not different between the two groups (Fig. 2g) [protrusions: *Sca1*^*2Q*/*2Q*^ (0.36 ± 0.02/μm) vs *Sca1*^*154Q*/*2Q*^ (0.30 ± 0.02/μm), *p* = 0.0343; spines: *Sca1*^*2Q*/*2Q*^ (0.33 ± 0.02/μm) vs *Sca1*^*154Q*/*2Q*^ (0.25 ± 0.01/μm), *p* = 0.0175; filopodia: *Sca1*^*2Q*/*2Q*^ (0.033 ± 0.004/μm) vs *Sca1*^*154Q*/*2Q*^ (0.044 ± 0.006/μm), *p* = 0.1621; unpaired *t*-test]. The number of spines as a percentage of the total protrusions was significantly lower in 8-week-old *Sca1*^*154Q*/*2Q*^ mice than in *Sca1*^*2Q*/*2Q*^ mice (Fig. 2h) [*Sca1*^*2Q*/*2Q*^ (90 ± 1%) vs *Sca1*^*154Q*/*2Q*^ (85 ± 2%), *p* = 0.0403; unpaired *t*-test], whereas that of filopodia was higher in 8-week-old *Sca1*^*154Q*/*2Q*^ mice than in *Sca1*^*2Q*/*2Q*^ mice (Fig. 2h) [*Sca1*^*2Q*/*2Q*^ (10 ± 1%) vs *Sca1*^*154Q*/*2Q*^ (15 ± 2%), *p* = 0.0403; unpaired *t*-test]. Both formation and elimination rates of spines in 8-week-old *Sca1*^*154Q*/*2Q*^ mice were significantly higher than those in *Sca1*^*2Q*/*2Q*^ mice (Fig. 2i) [formation rate: *Sca1*^*2Q*/*2Q*^ (2.7 ± 0.7%) vs *Sca1*^*154Q*/*2Q*^ (13 ± 2%), *p* < 0.0001; elimination rate: *Sca1*^*2Q*/*2Q*^ (2.0 ± 0.8%) vs *Sca1*^*154Q*/*2Q*^ (12 ± 4%), *p* = 0.0176; unpaired *t*-test]. The formation rate of filopodia in 8-week-old *Sca1*^*154Q*/*2Q*^ mice was higher than that in *Sca1*^*2Q*/*2Q*^, whereas the elimination rate was not significantly different (Fig. 2j) [formation rate: *Sca1*^*2Q*/*2Q*^ (5 ± 3%) vs *Sca1*^*154Q*/*2Q*^ (25 ± 8%), *p* = 0.0134; elimination rate: *Sca1*^*2Q*/*2Q*^ (21 ± 8% vs *Sca1*^*154Q*/*2Q*^ (42 ± 11%), *p* = 0.1166; unpaired *t*-test]. These data indicate that SCA1 mice have immature dendritic morphology and a loss of dendritic protrusions associated with persisting synaptic instability and the progression of SCA1 symptoms.

**Figure 3:**
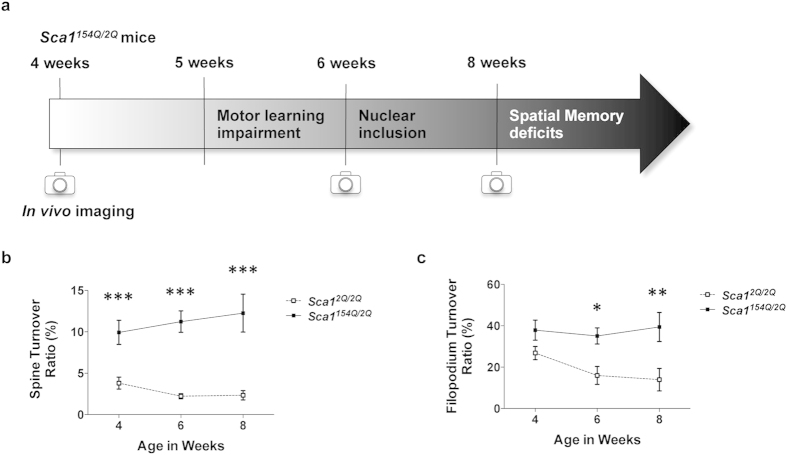
Dendritic protrusions do not stabilize with maturation in SCA1 mice. (**a**) Schematic of symptom progression in *Sca1*^*154Q*/*2Q*^ mice. (**b**) Compiled turnover rates of spines (ratio of spines formed and eliminated to twice the total number of spines) in 4-, 6-, and 8-week-old *Sca1*^*2Q*/*2Q*^ and *Sca1*^*154Q*/*2Q*^ mice. *Sca1*^*154Q*/*2Q*^ mice demonstrated higher spine turnover rates throughout synaptic development than *Sca1*^*2Q*/*2Q*^ mice. (**c**) Compiled turnover rates of filopodia at different ages (in weeks). *Sca1*^*154Q*/*2Q*^ mice showed higher filopodium turnover rates than *Sca1*^*2Q*/*2Q*^ mice from 6 weeks of age. Data are presented as the mean ± SEM. *p < 0.05, **p < 0.01, ***p < 0.001, Student *t*-test (**b**) or two-way ANOVA followed by Bonferroni test (**c**).

To evaluate protrusion stabilization that occurs with neuronal circuitry development and its disruption in SCA1 mice, we investigated the 1 h turnover rate of spines and filopodia in SCA1 mice at various ages during development (Fig. 3a). In *Sca1*^*154Q*/*2Q*^ mice, the spine turnover rate was significantly higher than that in *Sca1*^*2Q*/*2Q*^ mice throughout synaptic maturation (Fig. 3b; 4 wks: *p* = 0.0009; 6 wks: *p* < 0.0001; 8 wks: *p* < 0.0001; two-way ANOVA followed by Bonferroni test), whereas filopodium turnover rate was higher in 6- and 8-week-old *Sca1*^*154Q*/*2Q*^ mice (Fig. 3c; 4 wks: *p* = 0.2299; 6 wks: *p* = 0.0123; 8 wks: *p* = 0.0042; two-way ANOVA followed by Bonferroni test). No significant difference was observed in filopodium turnover rate at 4 weeks of age. This may be because of the high turnover rate of filopodia during early synaptic development, even in the control group. These results indicate that the normal development of dendritic protrusions, particularly filopodium stabilisation, is disrupted in *Sca1*^*154Q*/*2Q*^ mice.

### SCA1 mice exhibit progressive impairments in the density and morphology of dendritic protrusions in the hippocampus

**Figure 4:**
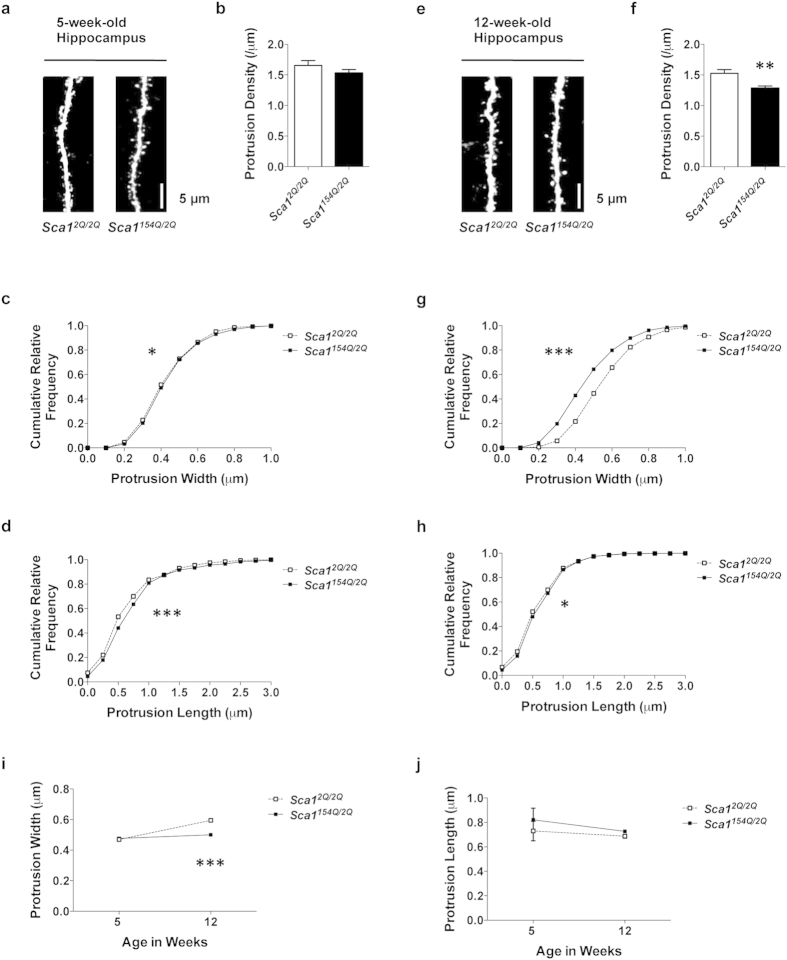
SCA1 mice show progressive deficits in the density and morphology of hippocampal dendritic protrusions. (**a–h**) Analysis of dendrites in the hippocampal CA1 stratum radiatum of 5- (**a–d**) and 12-week-old (**e–h**) *Sca1*^*2Q*/*2Q*^ mice (*n* = 15 dendrites from three animals and *n* = 20 dendrites from four animals, respectively) and *Sca1*^*154Q*/*2Q*^ mice (*n* = 15 dendrites from three animals and *n* = 25 dendrites from five animals, respectively). (**a**,**e**) Confocal images of dendrites in 5- and 12-week-old *Sca1*^*2Q*/*2Q*^ and *Sca1*^*154Q*/*2Q*^ mice. Images are best projections (3–7 optical sections, 0.43 μm apart). (**b**,**f**) Dendritic protrusion density in *Sca1*^*2Q*/*2Q*^ and *Sca1*^*154Q*/*2Q*^ mice. *Sca1*^*154Q*/*2Q*^ mice showed a lower protrusion density than *Sca1*^*2Q*/*2Q*^ mice at 12 weeks of age. (**c**,**g**) Cumulative frequency distribution of protrusion width in *Sca1*^*2Q*/*2Q*^ and *Sca1*^*154Q*/*2Q*^ mice. The distribution of protrusion width was abnormal in *Sca1*^*154Q*/*2Q*^ mice, particularly at 12 weeks of age. (**d**,**h**) Cumulative frequency distribution of protrusion length in *Sca1*^*2Q*/*2Q*^ and *Sca1*^*154Q*/*2Q*^ mice. The distribution of protrusion length was abnormal in *Sca1*^*154Q*/*2Q*^ mice at both ages. (**i**) Mean protrusion width at 5 and 12 weeks of age. *Sca1*^*154Q*/*2Q*^ mice had narrower protrusions than *Sca1*^*2Q*/*2Q*^ mice at 12 weeks of age. (**j**) Mean protrusion length at 5 and 12 weeks of age. *Sca1*^*154Q*/*2Q*^ mice had normal protrusion lengths at both ages. Data are presented as the mean ± SEM (**b**,**f**,**i**,**j**). *p < 0.05, **p < 0.01, ***p < 0.001, Student *t*-test (**b**,**f**), Kolmogorov–Smirnov test (**c**,**d**,**g**,**h**), or two-way ANOVA followed by Bonferroni test (**i**,**j**). Scale bar, 5 μm.

*Sca1*^*154Q*/*2Q*^ mice show spatial memory deficits at 8 weeks of age^5^, but there is little neuronal death in the hippocampus, a region associated with spatial learning^6^. To elucidate the neuronal dysfunction associated with cognitive deficit in the absence of neuronal loss, we focused on hippocampal CA1 dendrites and investigated the density and morphology of dendritic protrusions in SCA1 mice. We performed confocal laser-scanning microscopy on slices of fixed brain samples from 5- and 12-week-old *Sca1*^*2Q*/*2Q*^ and *Sca1*^*154Q*/*2Q*^ mice (Fig. 4a,e). We chose this technique because the hippocampus is deep within the brain, precluding the non-invasive use of *in vivo* two-photon imaging. No difference in protrusion density was observed between *Sca1*^*2Q*/*2Q*^ and *Sca1*^*154Q*/*2Q*^ mice at 5 weeks of age (Fig. 4b) [*Sca1*^*2Q*/*2Q*^ (1.66 ± 0.08/μm) vs *Sca1*^*154Q*/*2Q*^ (1.53 ± 0.06/μm), *p* = 0.2135; unpaired *t*-test], whereas at 12 weeks, dendritic protrusion density was significantly lower in *Sca1*^*154Q*/*2Q*^ mice than in *Sca1*^*2Q*/*2Q*^ mice (Fig. 4f) [*Sca1*^*2Q*/*2Q*^ (1.53 ± 0.06/μm) vs *Sca1*^*154Q*/*2Q*^ (1.28 ± 0.04/μm), *p* = 0.0011; unpaired *t*-test]. An abnormal frequency distribution of dendritic protrusion width was observed in *Sca1*^*154Q*/*2Q*^ mice, particularly at 12 weeks of age, when the distribution curve shifted to the left (Fig. 4c,g; 5 wks: *p* = 0.0107; 12 wks: *p* < 0.0001; Kolmogorov–Smirnov test). The mean dendritic protrusion width in 12-week-old *Sca1*^*154Q*/*2Q*^ mice was lower than that in *Sca1*^*2Q*/*2Q*^ mice (Fig. 4i; 5 wks: *p* = 0.8637; 12 wks: *p* < 0.0001; two-way ANOVA followed by Bonferroni test). These results show that the dendritic protrusion width in *Sca1*^*154Q*/*2Q*^ mice decreased as SCA1 symptoms developed. An abnormal frequency distribution of protrusion length in *Sca1*^*154Q*/*2Q*^ mice was evident at 5 weeks of age, and the frequency distribution curve was shifted to the right (Fig. 4d,h; 5 wks: *p* < 0.0001; 12 wks: *p* = 0.0441; Kolmogorov–Smirnov test). No difference was observed in the mean length of dendritic protrusions between *Sca1*^*154Q*/*2Q*^ and *Sca1*^*2Q*/*2Q*^ mice at either age (Fig. 4j; 5 wks: *p* = 0.5210; 12 wks: *p* > 0.9999; two-way ANOVA followed by Bonferroni test). These results indicate that the protrusion lengths in *Sca1*^*154Q*/*2Q*^ mice differ little from those in *Sca1*^*2Q*/*2Q*^ mice from early life through to adulthood. In summary, SCA1 mice demonstrated progressive deficits in dendritic protrusions in the hippocampus from adult ages.

### Decreased expression of synaptic scaffolding proteins in SCA1 mice

**Figure 5:**
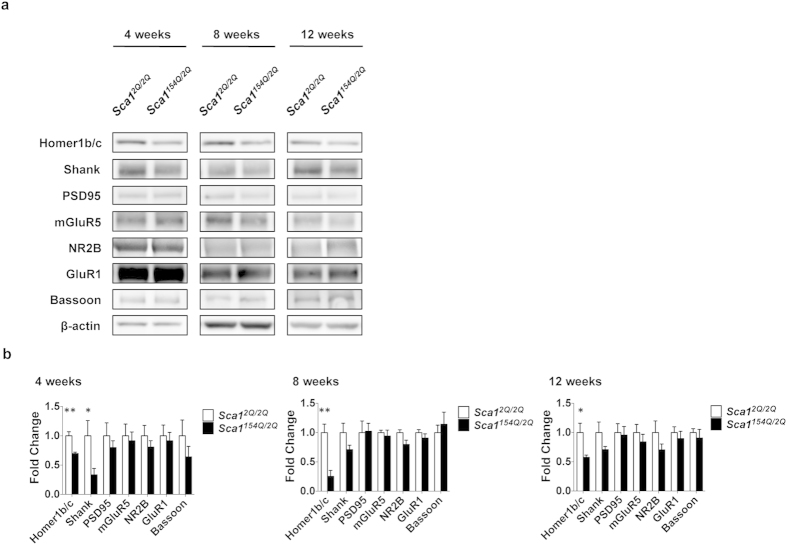
Low synaptic scaffolding protein expression in SCA1 mice. (**a**) Immunoblotting of the cerebral cortex in 4-, 8-, and 12-week old *Sca1*^*2Q*/*2Q*^ and *Sca1*^*154Q*/*2Q*^ mice. (**b**) Protein expression levels of *Sca1*^*2Q*/*2Q*^ and *Sca1*^*154Q*/*2Q*^ mice at 4 (left), 8 (middle), and 12 (right) weeks of age (*n* = 5 mice in all analyses). Data are presented as the mean ± SEM. *p < 0.05, **p < 0.01, Student *t*-test.

To investigate the molecular mechanism causing synaptic instability and subsequent SCA1 pathology, we measured the expression levels of synaptic proteins in the cerebral cortex by immunoblotting (Fig. 5a). Homer1b/c protein expression in *Sca1*^*154Q*/*2Q*^ mice was significantly lower at 4 (0.70 ± 0.07-fold; *p* = 0.0032; unpaired *t*-test), 8 (0.3 ± 0.2-fold; *p* = 0.0055; unpaired *t* test), and 12 weeks of age (0.6 ± 0.2-fold; *p* = 0.0330; unpaired *t*-test) than in age-matched *Sca1*^*2Q*/*2Q*^ mice (Fig. 5b). Shank protein levels were also lower in *Sca1*^*154Q*/*2Q*^ mice compared with *Sca1*^*2Q*/*2Q*^ mice at 4 weeks of age (0.3 ± 0.3-fold; *p* = 0.0466; unpaired *t*-test) (Fig. 5b). These results provide evidence that reduced expression of the synaptic scaffolding proteins Homer and Shank is associated with early synaptic pathology in SCA1 mice.

## Discussion

Understanding the mechanisms of the neuronal dysfunction that precedes behavioural impairments and neuronal death is a longstanding challenge in neurodegenerative diseases such as SCA1. We found that *Sca1*^*154Q*/*2Q*^ mice showed abnormal synaptic instability in the cerebral cortex during the development of neuronal circuitry, when apparent nuclear inclusions, neuronal death, or behavioural impairments are not yet observed. Synaptic instability in the cerebral cortex of *Sca1*^*154Q*/*2Q*^ mice persisted into adulthood in the cerebral cortex, and subsequent deficits in the number and morphology of dendritic protrusions became evident as symptoms developed. We also observed progressive deficits of dendritic protrusions in *Sca1*^*154Q*/*2Q*^ hippocampus, a region implicated in cognitive dysfunction in SCA1. Furthermore, compared with *Sca1*^*2Q*/*2Q*^ mice, *Sca1*^*154Q*/*2Q*^ mice showed lower expression levels of the postsynaptic scaffolding proteins Homer and Shank, even before synaptic maturation, when increased synaptic instability was observed. These results suggest that one of the mechanisms underlying neuronal dysfunction in SCA1 involves the association of synaptic instability, abnormal protrusion morphology during synaptic development, and a decline in scaffolding protein expression. We therefore hypothesized that impaired synaptic development triggers subsequent neurological symptoms and pathological abnormalities.

We used an established knock-in mouse model of SCA1, which expresses mutant ataxin-1 at endogenous levels in the normal spatial and temporal pattern, and accurately replicates pathological features observed in the human disease^5^,^13^. *Sca1*^*154Q*/*2Q*^ mice show motor learning impairment by approximately 5 weeks of age, which is followed by the development of nuclear inclusions, cognitive deficits, and Purkinje cell death. Other studies have also demonstrated motor learning impairment in 5-week-old mice, before neuronal death in the cerebellum, which occurs only in the late stages of the disease, using *Sca1* transgenic mice expressing full-length human *ATXN1* cDNAs with 82 CAG repeats specific to Purkinje cells^14^,^15^. Therefore, the abnormal synaptic instability detected in 4-week-old *Sca1*^*154Q*/*2Q*^ mice in the present study is one of the earliest pathological signs observed in SCA1 mouse models.

We performed *in vivo* imaging to rigorously evaluate the synaptic pathology of SCA1, because non-neuronal cells expressing mutant ataxin-1 are also involved in the pathogenesis of SCA1 models^16^,^17^,^18^. Furthermore, we believe that analysis of protrusion dynamics in living animals, in addition to morphology, enabled us to detect potential synaptic lesions. There have been cases in which changes in protrusion dynamics were observed, without any alterations in protrusion density, upon changes in synaptic plasticity and pathology^10^,^19^. Indeed, we also demonstrated a higher turnover rate of dendritic protrusions in 4-week-old *Sca1*^*154Q*/*2Q*^ mice than in *Sca1*^*2Q*/*2Q*^ mice, in the absence of any differences in protrusion density. We applied the thinned-skull method for *in vivo* imaging, which allows excitation and emission lights to penetrate the skull without eliciting any microglial inflammatory responses^20^, because many neurodegenerative diseases are associated with inflammation of the brain^21^,^22^.

A recent study using the rotarod test demonstrated that motor skill acquisition and coordination require the activation of neurons in the secondary motor cortex, which receives inputs from the somatosensory cortex^23^. Here, we demonstrated synaptic instability and dendritic spine loss in the somatosensory cortex of *Sca1*^*154Q*/*2Q*^ mice, which also show impaired rotarod performance^5^. These results suggest that *Sca1*^*154Q*/*2Q*^ mice have deficits in somatosensory and sensorimotor function. In the cerebellum of *Sca1*^*154Q*/*2Q*^ mice, however, no synaptic dysfunction, neuronal cell death or nuclear inclusions of ataxin-1 protein are observed when motor incoordination develops at 5 weeks of age^5^. *Sca1*^*154Q*/*2Q*^ mice do not show cerebellar neurodegeneration until 16 weeks and nuclear inclusions until 21 weeks. Therefore, the involvement of cerebellar dysfunction in the early symptomatic stage in *Sca1*^*154Q*/*2Q*^ mice remains unclear.

Previous studies, using conditional *Sca1* transgenic mice that stage-specifically express a full-length human *ATXN1* cDNA with 82 CAG repeats in the cerebellum, have demonstrated that the suppression of mutant ataxin-1 expression during the first 14 postnatal weeks inhibits subsequent motor dysfunction and dendritic atrophy^24^,^25^. Suppressing the expression of mutant ataxin-1, even for the first 5 postnatal weeks, can also inhibit impairments in synaptic transmission in the adult cerebellum^26^. Neuronal circuitry development occurs during the first few postnatal weeks, with a decrease in the turnover of dendritic protrusions and in the ratio of filopodia to total protrusions^9^. We identified enhanced synaptic instability in *Sca1*^*154Q*/*2Q*^ mice at 4 weeks of age compared with controls. These results suggest that the stage at which synaptic development occurs is a critical period in SCA1 pathogenesis, and that dendritic protrusions are excessively unstable in SCA1 mice and do not stabilise with maturation. Our present findings can be interpreted as developmental impairment in the synapses of SCA1 mice. This is a conceptually novel finding that implies that it is not only neurodevelopmental disorders, such as fragile X syndrome, autism spectrum disorder, and schizophrenia that involve deficits in synaptic development, but also SCA1, a late-onset neurodegenerative disease. It should be noted that synaptic instability in SCA1 mice is commonly observed in animal models of these neurodevelopmental disorders^19^,^27^,^28^,^29^. Interestingly, *Shank* genes, associated with autism, were also downregulated in SCA1 mice, and Shank is required for the maintenance of the density and morphology of dendritic spines^30^,^31^. Other studies using mouse models of neurodegenerative diseases such as Alzheimer’s and Huntington’s have also demonstrated instability and progressive loss of dendritic protrusions similar to that observed in *Sca1*^*154Q*/*2Q*^ mice^32^,^33^; however, these studies did not investigate the dynamics of dendritic protrusions at 4 weeks of age, i.e., during synaptic development. In contrast, we detected abnormal synaptic instability in 4-week-old *Sca1*^*154Q*/*2Q*^ mice. It is possible that the Alzheimer’s and Huntington’s disease models both show synaptic instability during development of neuronal circuitry, due to the similarities in synapse pathologies and subsequent progression of symptoms among these neurodegenerative disease models^34^,^35^. In Huntington’s disease models in particular, there are many similarities to *Sca1*^*154Q*/*2Q*^ mice: both Huntington’s and SCA1 are polyglutamine diseases; Huntington’s mouse models (R6/1 and R6/2) and *Sca1*^*154Q*/*2Q*^ mice have the same extent of expanded CAG repeats; and both models show synaptopathy^36^,^37^,^38^,^39^,^40^. We can therefore hypothesize that many neurodegenerative diseases share latent deficits in neuronal circuitry development, which precede the onset of symptoms.

The hippocampus of *Sca1*^*154Q*/*2Q*^ mice show impaired CA1 synaptic plasticity and dendritic arborization by 24 weeks of age, but no differences from control mice are observed until 8 weeks of age^5^,^13^. Our present results indicate that an evident decrease in the density of dendritic protrusions in the hippocampus of *Sca1*^*154Q*/*2Q*^ mice occurs between 5 and 12 weeks of age. This suggests that synaptic deficits in the hippocampus of *Sca1*^*154Q*/*2Q*^ mice develop by 12 weeks, and that they are mainly due to postsynaptic impairments. In the cerebral cortex, however, *Sca1*^*154Q*/*2Q*^ mice showed synaptic instability by 4 weeks of age and a decrease in synaptic number by 6 weeks. It is possible that in the hippocampus, *Sca1*^*154Q*/*2Q*^ mice also develop deficits in dendritic protrusion dynamics during the early stages of development. *Sca1*^*154Q*/*2Q*^ knock-in mice demonstrate cerebellar abnormalities, manifesting as motor learning impairment, at 5 weeks of age, although there is little difference from controls in the electrophysiological properties of Purkinje cells at this age^5^. The association between motor behavioural impairment and synaptic dysfunction in the cerebellum of *Sca1*^*154Q*/*2Q*^ mice remains unknown, and *in vivo* imaging studies of the synapses of Purkinje cells in *Sca1*^*154Q*/*2Q*^ mice are warranted.

In the present study, we found that expression levels of Homer and Shank proteins were lower in *Sca1*^*154Q*/*2Q*^ mice than in *Sca1*^*2Q*/*2Q*^ mice. This decline in the expression of postsynaptic scaffolding proteins occurred before synaptic maturation. Interestingly, Shank proteins were lower in *Sca1*^*154Q*/*2Q*^ mice exclusively at 4 weeks of age. *Shank1* and *Shank2* mRNA expression are higher during postnatal brain development than after maturation^41^, and their temporal expression patterns are similar to that of *ATXN1* mRNA^42^. Therefore, the effect of mutant ataxin-1 on the expression of Shank proteins may be strongest during postnatal development. These lines of evidence provide an insight into the molecular mechanisms of developmental impairments in the synapses of *Sca1*^*154Q*/*2Q*^ mice. It is interesting that Shank1 knock-out mice show impaired rotarod performance and decreased spine width, similarly to *Sca1*^*154Q*/*2Q*^ mice^43^. Homer and Shank, which form a polymeric network structure at postsynaptic sites, interact with glutamate receptors and regulate their downstream signalling, inducing the accumulation of inositol-1,4,5-triphosphate (IP3) receptors in protrusions^44^,^45^,^46^. Moreover, Homer and Shank proteins regulate the morphology and function of dendritic protrusions^31^,^45^. Because the morphology of dendritic protrusions correlates with the dynamics of these proteins^47^, Homer and Shank proteins may be involved in protrusion turnover. Our results are supported by previous studies that demonstrated a reduction in the levels of Homer3 and IP3 receptors in the Purkinje cells of *Sca1* transgenic mice^48^,^49^. Taken together, this evidence suggests that *Sca1*^*154Q*/*2Q*^ mice have deficits in Homer- and Shank-mediated intracellular calcium release from IP3 receptors, and the deficits may cause synaptic instability and abnormal synaptic maturation.

## Materials and Methods

All experimental protocols were approved by an Animal Ethics Committee at the National Institute of Neuroscience, National Center of Neurology and Psychiatry, Japan, and performed in strict accordance with institutional guidelines.

### Experimental animals

*Thy1-YFP* H-line mice, expressing yellow fluorescent protein (YFP) predominantly in layer 5 pyramidal neurons, were purchased from the Jackson Laboratory^50^. *Sca1*^*154Q*/*2Q*^ mice were kindly provided by Dr. K. Watase at Tokyo Medical and Dental University^5^. Both mouse strains were backcrossed to C57BL/6J mice. Heterozygous *Thy1-YFP* mice were crossed with *Sca1*^*154Q*/*2Q*^ mice to yield double transgenic animals heterozygous for *Thy1-YFP* and the knock-in mutation of the *Sca1* allele (*Thy1-YFP*^*+*/*−*^; *Sca1*^*154Q*/*2Q*^). As a control, age-matched littermates heterozygous for *Thy1-YFP* (*Thy1-YFP*^*+*/*−*^; *Sca1*^*2Q*/*2Q*^) were used. Only male offspring heterozygous for YFP were used because the density of dendritic spines in female mice fluctuates daily due to the oestrus cycle^51^. *Thy1-YFP*^*+*/*−*^; *Sca1*^*2Q*/*2Q*^ mice are described as *Sca1*^*2Q*/*2Q*^ mice, and *Thy1-YFP*^*+*/*−*^; *Sca1*^*154Q*/*2Q*^ mice are described as *Sca1*^*154Q*/*2Q*^ mice throughout the manuscript. Mice were housed four per cage under controlled temperature (25 ± 1 °C) and lighting (12 h light/dark cycle), and were provided with food and water *ad libitum*.

### Surgical procedure for *in vivo* imaging

The thinned-skull cranial window technique^52^ was used because it is less invasive than the open-skull method^20^. *Sca1*^*2Q*/*2Q*^ and *Sca1*^*154Q*/*2Q*^ mice expressing YFP were deeply anesthetized with intraperitoneal ketamine and xylazine (0.1 and 0.015 mg/g body weight, respectively). Body temperature was maintained at 37 °C with a heating pad during surgery and imaging. Eyes were lubricated with ointment to prevent dryness. After scalp incision, the primary somatosensory area (1.1 mm posterior to bregma and 3.4 mm lateral from the midline) was identified with stereotactic coordinates. A small metal plate with a round hole was glued onto the skull with cyanoacrylate glue and acrylic resin dental cement (Unifast; GC, Tokyo, Japan), and mice were fixed to a custom-made skull immobilization stage via the metal plate. The skull above the imaging area, located in the center of the hole in the metal plate, was thinned to approximately 20 μm with a high-speed microdrill (UG23A/UC210C; Urawa, Saitama, Japan) and microsurgical blade (USM-6400; Sable Industries, Vista, CA, USA). The hole in the metal plate was filled with artificial cerebrospinal fluid during surgery and imaging.

### *In vivo* transcranial two-photon imaging

*Sca1*^*2Q*/*2Q*^ and *Sca1*^*154Q*/*2Q*^ mice were imaged under anaesthesia using a two-photon laser-scanning microscope (FV1000-MPE; Olympus, Japan) with a water-immersion objective lens (25×, NA 1.05) at 8× digital zoom, yielding high-magnification images suitable for the quantification of dendritic spines. A Ti-sapphire laser (MaiTai HP DeepSee-OL; Spectra-Physics, Mountain View, CA, USA) was tuned to 950 nm. To minimize phototoxicity, laser intensity was maintained between 10 and 30 mW at the focus. Image stacks (512 × 512 pixels; 0.124 μm/pixels; 0.75 μm z-step) were taken at approximately 70 μm below the pial surface, where layer 1 dendrites of layer 5 pyramidal neurons are located. Dendrites were imaged for each experiment at time 0, and again after an interval of either 1 or 48 h. Image acquisition time in each imaging session was approximately 5 minutes. In the 1 h interval experiment, mice were maintained under anaesthesia between the two imaging sessions. In the 48 h interval experiments, mice were allowed to recover from anaesthesia after the first imaging session and were returned to their home cages until the next session.

### Confocal laser-scanning microscopy for *ex vivo* fixed samples

YFP-labelled *Sca1*^*2Q*/*2Q*^ and *Sca1*^*154Q*/*2Q*^ mice were anesthetized and perfused transcardially with phosphate-buffered saline (pH 7.4) followed by 4% paraformaldehyde. Brains were removed and 50 μm sections were cut with a Vibratome 3000 (Vibratome Company, St Louis, MO, USA). Image stacks (1024 × 1024 pixels; 0.069 μm/pixel; 0.43 μm z-step) were taken of the secondary dendrites of CA1 neurons in the dorsal hippocampus, using a confocal laser-scanning microscope (FV1000; Olympus, Japan) with a silicone-immersion objective lens (60×, NA 1.3) at a digital zoom of 3. For YFP, the excitation and emission wavelengths were 488 nm and 515 nm, respectively.

### Image analysis

The turnover rate, density, head width, and neck length of the postsynaptic dendritic protrusions were analysed with Neurolucida neuron tracing software (MicroBrightField, Williston, VT, USA) from three-dimensional two-photon or confocal z-stacks. Morphometric analysis of dendritic protrusions was performed in accordance with a previous report^9^. Filopodia were identified as long, thin structures (head width to neck width ratio <1.2:1; protrusion length to neck width ratio >3:1). Other protrusions were classified as spines. Protrusions were identified as being identical between two successive frames by their spatial relationship to adjacent landmarks (e.g., axonal and dendritic orientations) and their relative position to immediately adjacent protrusions. Protrusions were considered different between two successive frames if they were located >0.7 μm from their expected positions based on the first image. The formation and elimination rates of the protrusions were defined as the percentage of protrusions that appeared and disappeared, respectively, between two successive frames, relative to the total protrusion number. Protrusion turnover rate was defined as the sum of the protrusions formed and eliminated, divided by twice the total number of protrusions. Data were collected from 10–18 dendrites and 460–1,162 protrusions in four to eight mice for *in vivo* studies, and from 15–25 dendrites and 1,676–2,350 protrusions in three to five mice for *ex vivo* studies.

### Immunoblotting

Tissues were lysed in RIPA buffer [50 mM Tris (pH 7.4), 1% Nonidet P-40, 0.25% sodium deoxycholate, 150 mM NaCl, 1 mM EDTA] with EDTA-free complete protease inhibitors and PhosSTOP (Roche Applied Science). Immunoblotting was performed using the following antibodies: anti-Homer1b/c (~47 kDa; Cat. #160 023, Synaptic Systems), anti-pan-Shank (~159 kDa; MABN24, EMD Millipore), anti-PSD95 (~100 kDa; ab13552, Abcam), anti-mGluR5 (~132 kDa; AB5675, EMD Millipore), anti-NR2B (~166 kDa; MAB5220, EMD Millipore), anti-GluR1 (~106 kDa; 06-306-MN; Upstate), anti-Bassoon (~420 kDa; 6897S; Cell Signaling Technology), and anti-β-actin (~42 kDa; A5441; Sigma-Aldrich). Total protein (10 μg/lane) was separated on sodium dodecyl sulphate-polyacrylamide gels, transferred to immunoblot polyvinylidene difluoride membranes (Bio-Rad, Hercules, CA), incubated with 3% BSA in TBST [50 mM Tris (pH 7.0), 150 mM NaCl, 0.05% Tween 20] for 1 h at room temperature, and incubated with each primary antibody overnight at 4 °C. The membranes were washed in TBST and further incubated with anti-mouse/rabbit IgG-horseradish peroxidase conjugate (Cat. #31430 and 31460, respectively; Thermo Scientific Pierce Antibodies). After washing in TBST, the membranes were developed with Super Signal West Dura or Femto extended duration substrate (Pierce) and analysed using a ChemiImager (Alpha Innotech, San Leandro, CA). Proteins were identified by band position relative to a molecular weight marker.

### Statistical analysis

To determine statistical significance, we used the Student *t*-test, one-way ANOVA followed by Tukey multiple-comparison test, two-way ANOVA followed by Bonferroni multiple-comparison test, or the Kolmogorov–Smirnov test. All statistical analyses were performed with GraphPad Prism (GraphPad Software Inc, La Jolla, CA, USA). All data are presented as the mean ± SEM.

## Additional Information

**How to cite this article**: Hatanaka, Y. *et al.* Abnormalities in synaptic dynamics during development in a mouse model of spinocerebellar ataxia type 1. *Sci. Rep.* **5**, 16102; doi: 10.1038/srep16102 (2015).

## References

[b1] Taylor J. P., Hardy J. & Fischbeck K. H. Toxic proteins in neurodegenerative disease. Science 296, 1991–1995 (2002). 12065827 10.1126/science.1067122

[b2] Bredesen D. E., Rao R. V. & Mehlen P. Cell death in the nervous system. Nature 443, 796–802 (2006). 17051206 10.1038/nature05293 PMC3970704

[b3] Palop J. J., Chin J. & Mucke L. A network dysfunction perspective on neurodegenerative diseases. Nature 443, 768–773 (2006). 17051202 10.1038/nature05289

[b4] Orr H. T. *et al.* Expansion of an unstable trinucleotide CAG repeat in spinocerebellar ataxia type 1. Nat Genet 4, 221–226 (1993). 8358429 10.1038/ng0793-221

[b5] Watase K. *et al.* A long CAG repeat in the mouse Sca1 locus replicates SCA1 features and reveals the impact of protein solubility on selective neurodegeneration. Neuron 34, 905–919 (2002). 12086639 10.1016/s0896-6273(02)00733-x

[b6] Zoghbi H. Y. & Orr H. T. Spinocerebellar ataxia type 1. Semin Cell Biol 6, 29–35 (1995). 7620119 10.1016/1043-4682(95)90012-8

[b7] Orr H. T. & Zoghbi H. Y. Trinucleotide repeat disorders. Annu Rev Neurosci 30, 575–621 (2007). 17417937 10.1146/annurev.neuro.29.051605.113042

[b8] Lorenzetti D. *et al.* Repeat instability and motor incoordination in mice with a targeted expanded CAG repeat in the Sca1 locus. Hum Mol Genet 9, 779–785 (2000). 10749985 10.1093/hmg/9.5.779

[b9] Grutzendler J., Kasthuri N. & Gan W. B. Long-term dendritic spine stability in the adult cortex. Nature 420, 812–816 (2002). 12490949 10.1038/nature01276

[b10] Trachtenberg J. T. *et al.* Long-term *in vivo* imaging of experience-dependent synaptic plasticity in adult cortex. Nature 420, 788–794 (2002). 12490942 10.1038/nature01273

[b11] Penzes P., Cahill M. E., Jones K. A., VanLeeuwen J. E. & Woolfrey K. M. Dendritic spine pathology in neuropsychiatric disorders. Nat Neurosci 14, 285–293 (2011). 21346746 10.1038/nn.2741 PMC3530413

[b12] Yang G., Chang P. C., Bekker A., Blanck T. J. & Gan W. B. Transient effects of anesthetics on dendritic spines and filopodia in the living mouse cortex. Anesthesiology 115, 718–726 (2011). 21768874 10.1097/ALN.0b013e318229a660 PMC3815535

[b13] Watase K. *et al.* Lithium therapy improves neurological function and hippocampal dendritic arborization in a spinocerebellar ataxia type 1 mouse model. PLoS Med 4, e182 (2007). 17535104 10.1371/journal.pmed.0040182 PMC1880853

[b14] Burright E. N. *et al.* SCA1 transgenic mice: a model for neurodegeneration caused by an expanded CAG trinucleotide repeat. Cell 82, 937–948 (1995). 7553854 10.1016/0092-8674(95)90273-2

[b15] Clark H. B. *et al.* Purkinje cell expression of a mutant allele of SCA1 in transgenic mice leads to disparate effects on motor behaviors, followed by a progressive cerebellar dysfunction and histological alterations. J Neurosci 17, 7385–7395 (1997). 9295384 10.1523/JNEUROSCI.17-19-07385.1997 PMC6573461

[b16] Vig P. J. *et al.* Glial S100B Positive Vacuoles In Purkinje Cells: Earliest Morphological Abnormality In SCA1 Transgenic Mice. J Neurol Sci Turk 23, 166–174 (2006). 18176630 10.1901/jaba.2006.23-166 PMC2174790

[b17] Giovannoni R. *et al.* Reactive astrocytosis and glial glutamate transporter clustering are early changes in a spinocerebellar ataxia type 1 transgenic mouse model. Neuron Glia Biol 3, 335–351 (2007). 18634565 10.1017/S1740925X08000185

[b18] Shiwaku H. *et al.* Suppression of the novel ER protein Maxer by mutant ataxin-1 in Bergman glia contributes to non-cell-autonomous toxicity. EMBO J 29, 2446–2460 (2010). 20531390 10.1038/emboj.2010.116 PMC2910266

[b19] Cruz-Martin A., Crespo M. & Portera-Cailliau C. Delayed stabilization of dendritic spines in fragile X mice. J Neurosci 30, 7793–7803 (2010). 20534828 10.1523/JNEUROSCI.0577-10.2010 PMC2903441

[b20] Xu H. T., Pan F., Yang G. & Gan W. B. Choice of cranial window type for *in vivo* imaging affects dendritic spine turnover in the cortex. Nat Neurosci 10, 549–551 (2007). 17417634 10.1038/nn1883

[b21] Morris G. P., Clark I. A., Zinn R. & Vissel B. Microglia: a new frontier for synaptic plasticity, learning and memory, and neurodegenerative disease research. Neurobiol Learn Mem 105, 40–53 (2013). 23850597 10.1016/j.nlm.2013.07.002

[b22] Cunningham C. Microglia and neurodegeneration: the role of systemic inflammation. Glia 61, 71–90 (2013). 22674585 10.1002/glia.22350

[b23] Cao V. Y. *et al.* Motor Learning Consolidates Arc-Expressing Neuronal Ensembles in Secondary Motor Cortex. Neuron 86, 1385–1392 (2015). 26051420 10.1016/j.neuron.2015.05.022 PMC4474764

[b24] Serra H. G. *et al.* RORalpha-mediated Purkinje cell development determines disease severity in adult SCA1 mice. Cell 127, 697–708 (2006). 17110330 10.1016/j.cell.2006.09.036

[b25] Zu T. *et al.* Recovery from polyglutamine-induced neurodegeneration in conditional SCA1 transgenic mice. J Neurosci 24, 8853–8861 (2004). 15470152 10.1523/JNEUROSCI.2978-04.2004 PMC6729947

[b26] Barnes J. A. *et al.* Abnormalities in the climbing fiber-Purkinje cell circuitry contribute to neuronal dysfunction in ATXN1[82Q] mice. J Neurosci 31, 12778–12789 (2011). 21900557 10.1523/JNEUROSCI.2579-11.2011 PMC3178465

[b27] Pan F., Aldridge G. M., Greenough W. T. & Gan W. B. Dendritic spine instability and insensitivity to modulation by sensory experience in a mouse model of fragile X syndrome. Proc Natl Acad Sci USA 107, 17768–17773 (2010). 20861447 10.1073/pnas.1012496107 PMC2955121

[b28] Isshiki M. *et al.* Enhanced synapse remodelling as a common phenotype in mouse models of autism. Nature communications 5, 4742 (2014). 10.1038/ncomms5742 25144834

[b29] Hayashi-Takagi A. *et al.* PAKs inhibitors ameliorate schizophrenia-associated dendritic spine deterioration *in vitro* and *in vivo* during late adolescence. Proc Natl Acad Sci USA 111, 6461–6466 (2014). 24706880 10.1073/pnas.1321109111 PMC4035976

[b30] Yoo J., Bakes J., Bradley C., Collingridge G. L. & Kaang B. K. Shank mutant mice as an animal model of autism. Philosophical transactions of the Royal Society of London. Series B, Biological sciences 369, 20130143 (2014). 24298145 10.1098/rstb.2013.0143 PMC3843875

[b31] Roussignol G. *et al.* Shank expression is sufficient to induce functional dendritic spine synapses in aspiny neurons. J Neurosci 25, 3560–3570 (2005). 15814786 10.1523/JNEUROSCI.4354-04.2005 PMC6725374

[b32] Tsai J., Grutzendler J., Duff K. & Gan W. B. Fibrillar amyloid deposition leads to local synaptic abnormalities and breakage of neuronal branches. Nat Neurosci 7, 1181–1183 (2004). 15475950 10.1038/nn1335

[b33] Murmu R. P., Li W., Holtmaat A. & Li J. Y. Dendritic spine instability leads to progressive neocortical spine loss in a mouse model of Huntington’s disease. J Neurosci 33, 12997–13009 (2013). 23926255 10.1523/JNEUROSCI.5284-12.2013 PMC6619731

[b34] Yu W. & Lu B. Synapses and dendritic spines as pathogenic targets in Alzheimer’s disease. Neural Plast 2012, 247150 (2012). 22474602 10.1155/2012/247150 PMC3306944

[b35] Nithianantharajah J. & Hannan A. J. Dysregulation of synaptic proteins, dendritic spine abnormalities and pathological plasticity of synapses as experience-dependent mediators of cognitive and psychiatric symptoms in Huntington’s disease. Neuroscience 251, 66–74 (2013). 22633949 10.1016/j.neuroscience.2012.05.043

[b36] Li J. Y., Popovic N. & Brundin P. The use of the R6 transgenic mouse models of Huntington’s disease in attempts to develop novel therapeutic strategies. NeuroRx: the journal of the American Society for Experimental NeuroTherapeutics 2, 447–464 (2005). 16389308 10.1602/neurorx.2.3.447 PMC1144488

[b37] Bulley S. J., Drew C. J. & Morton A. J. Direct Visualisation of Abnormal Dendritic Spine Morphology in the Hippocampus of the R6/2 Transgenic Mouse Model of Huntington’s Disease. Journal of Huntington’s disease 1, 267–273 (2012). 10.3233/JHD-120024 25063335

[b38] Klapstein G. J. *et al.* Electrophysiological and morphological changes in striatal spiny neurons in R6/2 Huntington’s disease transgenic mice. Journal of neurophysiology 86, 2667–2677 (2001). 11731527 10.1152/jn.2001.86.6.2667

[b39] Spires T. L. *et al.* Dendritic spine pathology and deficits in experience-dependent dendritic plasticity in R6/1 Huntington’s disease transgenic mice. Eur J Neurosci 19, 2799–2807 (2004). 15147313 10.1111/j.0953-816X.2004.03374.x

[b40] Nithianantharajah J., Barkus C., Vijiaratnam N., Clement O. & Hannan A. J. Modeling brain reserve: experience-dependent neuronal plasticity in healthy and Huntington’s disease transgenic mice. The American journal of geriatric psychiatry: official journal of the American Association for Geriatric Psychiatry 17, 196–209 (2009). 19454847 10.1097/JGP.0b013e318196a632

[b41] Bockers T. M. *et al.* Differential expression and dendritic transcript localization of Shank family members: identification of a dendritic targeting element in the 3' untranslated region of Shank1 mRNA. Molecular and cellular neurosciences 26, 182–190 (2004). 15121189 10.1016/j.mcn.2004.01.009

[b42] Banfi S. *et al.* Cloning and developmental expression analysis of the murine homolog of the spinocerebellar ataxia type 1 gene (Sca1). Hum Mol Genet 5, 33–40 (1996). 8789437 10.1093/hmg/5.1.33

[b43] Hung A. Y. *et al.* Smaller dendritic spines, weaker synaptic transmission, but enhanced spatial learning in mice lacking Shank1. J Neurosci 28, 1697–1708 (2008). 18272690 10.1523/JNEUROSCI.3032-07.2008 PMC2633411

[b44] Hayashi M. K. *et al.* The postsynaptic density proteins Homer and Shank form a polymeric network structure. Cell 137, 159–171 (2009). 19345194 10.1016/j.cell.2009.01.050 PMC2680917

[b45] Sala C. *et al.* Regulation of dendritic spine morphology and synaptic function by Shank and Homer. Neuron 31, 115–130 (2001). 11498055 10.1016/s0896-6273(01)00339-7

[b46] Tu J. C. *et al.* Coupling of mGluR/Homer and PSD-95 complexes by the Shank family of postsynaptic density proteins. Neuron 23, 583–592 (1999). 10433269 10.1016/s0896-6273(00)80810-7

[b47] Yasumatsu N., Matsuzaki M., Miyazaki T., Noguchi J. & Kasai H. Principles of long-term dynamics of dendritic spines. J Neurosci 28, 13592–13608 (2008). 19074033 10.1523/JNEUROSCI.0603-08.2008 PMC2706274

[b48] Lin X., Antalffy B., Kang D., Orr H. T. & Zoghbi H. Y. Polyglutamine expansion down-regulates specific neuronal genes before pathologic changes in SCA1. Nat Neurosci 3, 157–163 (2000). 10649571 10.1038/72101

[b49] Serra H. G. *et al.* Gene profiling links SCA1 pathophysiology to glutamate signaling in Purkinje cells of transgenic mice. Hum Mol Genet 13, 2535–2543 (2004). 15317756 10.1093/hmg/ddh268

[b50] Feng G. *et al.* Imaging neuronal subsets in transgenic mice expressing multiple spectral variants of GFP. Neuron 28, 41–51 (2000). 11086982 10.1016/s0896-6273(00)00084-2

[b51] Woolley C. S. & McEwen B. S. Estradiol mediates fluctuation in hippocampal synapse density during the estrous cycle in the adult rat. J Neurosci 12, 2549–2554 (1992). 1613547 10.1523/JNEUROSCI.12-07-02549.1992 PMC6575846

[b52] Yang G., Pan F., Parkhurst C. N., Grutzendler J. & Gan W. B. Thinned-skull cranial window technique for long-term imaging of the cortex in live mice. Nat Protoc 5, 201–208 (2010). 20134419 10.1038/nprot.2009.222 PMC4690457

